# Powered Air Purifying Respirators (PAPRs) for Protection from Respirable Dust in Underground Mines

**DOI:** 10.1007/s42461-025-01356-4

**Published:** 2025-09-20

**Authors:** Luis Sanchez Gonzalez, Ashish Ranjan Kumar, Barbara Arnold

**Affiliations:** Department of Energy and Mineral Engineering, The Pennsylvania State University, 58 Pollock Rd, University Park, PA 16802 USA

**Keywords:** Respirators, Coal dust, Simulations, Filtration, CFD

## Abstract

Underground mining operations use several remedial measures to alleviate the miners’ exposure to respirable dust. This includes maintaining the ventilation airflow, deploying scrubbers on equipment, and using water sprays to move air and dust away from the miners and to capture them. Despite these engineering controls, recent research shows an increased occurrence of exposure-related issues in the impacted miners. Masks and other PPE devices are considered the least preferred in the hierarchy of controls. However, they show a high protection factor if designed properly and according to recommendations. A Powered Air Purifying Respirator (PAPR) is a battery-operated personal scrubber that has found widespread application in industries. This respirator uses a blower to move air through a high-efficiency particulate air (HEPA) filter, delivering the purified air to the user. Its popularity is attributed to its high protection efficiency. This paper summarizes the current applications and evaluation methods of PAPRs. It strongly recommends their usage in underground mines to reduce the risk of mine dust lung diseases such as pneumoconiosis, asbestosis, silicosis, and others that do not have any conclusive treatment. While the high efficiency of the respirators has been demonstrated, we recommend further studies to investigate the unique challenges associated with their use in underground mines. Therefore, this paper also presents computational fluid dynamics (CFD) simulations as a tool to understand the performance of PAPRs in underground tunnels, which could help to understand not only the efficiency, but also the challenges associated with their implementation.

## Introduction

Almost all processes in a mining operation generate dust. These include mechanical extraction of the minerals, transportation, crushing, grinding, and others [1–3]. Dust particles in a surface mine are transported away from the source due to their small size and severely impact the vegetation, sources of water, and other crucial components of the surrounding environment [4, 5]. The consequences of dust particles in underground mines, especially coal operations, are much more severe due to their impact on the health of the exposed workers and the safety of the facilities. Mining operations use several remedial measures such as ventilation air, water sprays, scrubbers, and physical barriers to combat dust and to lower the miners’ exposure [6].

**Fig. 1:**
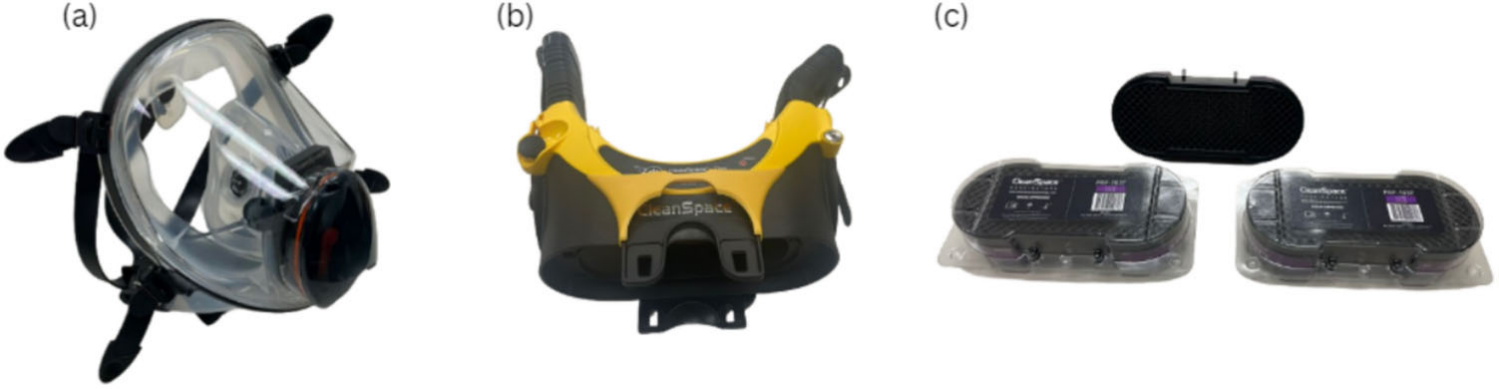
PAPR basic elements: **a** mask, **b** motor blower, **c** filtering elements. Elements shown correspond to a CleanSpace PAPR

Several factors control the dust particles’ toxicity. Out of these, particle size, constituents, and surface properties are the most significant [7]. For example, the size of the dust particles depends on the parent material and the extraction process. Generally, particles of an aerodynamic diameter smaller than 10.0 $\mu$m and a median cut-off size (d_50_) less than 4.0 $\mu$m are considered respirable [8, 9]. These particles travel deep inside the human lung, affect the oxygen-exchange mechanism of the alveoli, and impede nominal breathing. Despite better engineering controls in modern-day underground mines, recent reports have shown an increased occurrence of exposure-related ailments such as coal workers’ pneumoconiosis (CWP) and progressive massive fibrosis. Recent research in the central Appalachian region indicated that about 20.6% of the long-tenure coal miners have signs of CWP [10, 11]. Therefore, additional measures are required to alleviate their exposure.

Respirators are usually required when there is a significant concentration of contaminants (above the PEL). A Powered Air Purifying Respirator (PAPR) is a battery-operated respirator that uses a blower to force contaminated air through a HEPA filter, delivering clean air to the user [12]. These respirators provide better protection compared to traditional ones, which include negative-pressure respirators and elastomeric respirators with half-face and full-face masks. This is not only due to the higher efficiency of its filters, but also the positive pressure generated inside of the mask that prevents contaminants from leaking in [13]. They are used in construction, firefighting, agriculture, and other industries where the workers are exposed to respirable particulates. PAPRs are more expensive compared to disposable filters owing to their complex setup and components. Figure 1 shows their basic components. The constituent elements differ depending on the models and their applications; however, the ones shown below are found in all of them. This paper summarizes PAPRs, their protection factors, applications in other industries, computational fluid dynamics (CFD) modeling, industrial standards, experiment-based studies, and makes a strong case for the widespread use of PAPRs in underground mines.

## PAPR Protection Factors

Most respirators use fibrous filters as their main dust-capturing element. These filters trap the particulates on several layers at unique depths in their volume. This lowers the particulate concentration as the air passes through the filter. PAPR’s performance is quantified in terms of the decrease in the aerosol concentration downstream of the filter compared to the upstream concentration. A general definition of the protection factor is shown in Eq. 1 below.

1 $$\begin{aligned} Protection\ Factor=\frac{C_o}{C_i} \end{aligned}$$

where $C_o$ is the contaminant concentration outside the respirator and $C_i$ is the concentration inside it. Although these factors, in general, are calculated using similar methodologies, unique names are assigned to them based on conditions set during the measurement. Unique protection factors used commonly in the industry are described below:

- **Assigned protection factor (APF)**: The Occupational Safety and Health Organization (OSHA) defines the APF as the workplace level of respiratory protection that a respirator is expected to provide when used. To achieve the APF reported by the entities, the American National Standard for Respiratory Protection published in 1992 states that the respirator must be functional and should not have any defects. Additionally, the respirator must fit the user who must also be trained in its proper usage [14, 15].
- **Workplace protection factor (WPF)**: The workplace protection factor (WPF) refers to the expected protection level at specific work conditions [15]. This factor can be measured under simulated conditions (SWPF) or real working conditions (WPF). Several studies are focused on measuring the WPF as it allows companies to understand the protection provided for their specific field, considering the characteristics of the contaminants that are present. These studies will be addressed in Section 5.3 in this article [16–19].
- **Fit factor (FF)**: The fit factor (FF) is a measure of the protection factor of a specific respirator for a particular individual [15]. Even though the tight-fitting PAPRs may have a higher APF, it is necessary to conduct a fit test to ensure the desirable protection level is achieved. As the FF depends on several factors and varies between subjects, APF is assigned the value of the minimum FF reported in at least 95% of the subjects.

Literature shows significant differences between the APFs reported by different agencies. Initial efforts to establish the APF of respirators were made during 1969–1972 and were based on quantitative fit testing conducted at Los Alamos National Laboratory. APF values of 1000 were recommended for the PAPRs. However, these APFs were not determined systematically. The APF suggested for PAPRs was obtained by extrapolating data obtained from the performance of a self-contained breathing apparatus (SCBA) [20]. NIOSH recommended updating the values of APF with the WPFs whenever available [21]. Currently, NIOSH suggests an APF of 25 for any PAPR equipped with a hood or helmet and a HEPA filter [22].

It is important to note that respirators, including PAPRs, have tight-fitting designs to provide particulate-free air to the user and to realize the highest possible protection to the user. These respirators come in different sizes, and a fit test should be conducted to ensure the size is appropriate for the worker. Therefore, for users with facial hair or other conditions affecting the results from the fit test, a loose-fitting PAPR may be a better option [23]. Several studies have used manikins to calculate the manikin fit factor (mFF) [24, 25]. Based on the assigned protection factors, PAPRs can be classified as (i) loose-fitting and (ii) tight-fitting. Face-piece, hoods, and helmets are usually considered loose-fitting units. Full and half-face masks are categorized under the tight-fitting PAPRs. The APF value of tight-fitting respirators could be as low as 1000, while that of loose-fitting respirators is usually higher than 25. Tight-fitting masks offer better protection.

## Applications of PAPRs

During the last few years, the use of PAPRs has increased significantly. This is attributed to their high protection factors, comfort to the user, ease of donning, and carrying underground. This section summarizes their applications in different industries, advantages, and drawbacks.

**Table 1:** Coal dust $d_{50}$ at different MSHA Health and Safety Districts [34]

District	No. of mines	D (SD) ($\mu$m)
2	5	161 ± 23
3	7	149 ± 50
4	3	148 ± 33
5	4	134 ± 42
6	4	155 ± 35
7	4	126 ± 51
8	3	169 ± 42
9	4	160 ± 25
10	4	151 ± 25
11	6	116 ± 40

### Mining Industry

Dust of different sizes and characteristics is generated in mines. The mass median particle diameters recorded in ten MSHA districts are shown in Table 1. Other studies found particles with sizes of under 5 $\mu$m for silica and under 20 $\mu$m for asbestos fibers [26, 27]. Their inhalation has a detrimental impact on the exposed miners. Therefore, in addition to the remedial measures adopted by the mining operations, the enforcement agencies regulate several aspects of the underground mine infrastructure, procedures, and the environment to lower the exposure. For example, the “New Dust Rules” were enacted in 2014, which lowered the permissible coal dust concentration to 1.5 mg/m^3^ [28]. In 2024, the Silica Final Rule was released, which lowered the PEL of respirable silica to 50 $\mu$g/m^3^. This new regulation also included control methods and timelines for compliance [29].

PAPRs are not new to the mining industry. One of the first uses of PAPRs was to protect uranium miners from daughter products of radon in the 1960s. The experiments demonstrated that PAPRs offer protection factors of up to 1000 under difficult work conditions, much higher than the protection factor of 20 required at that time [30]. A Ukrainian study on negative-pressure half masks showed dust concentrations inside the mask between 8.6 and 24.7 mg/m^3^, which is higher than the permissible exposure limit (PEL), making PAPRs an attractive option. A study in Australia showed that PAPRs can reach higher protection factors than the established APF (25); the results are shown in Table 2 [31, 32].

**Table 2:** PAPRs used in the Australian mining industry

Supplier	PAPR	Filter	WPF
3M Australia	3M^™^ Airstream^™^	060-23-11PAUS (P2 rated*)	50
Dräger Safety Australia	Dräger X-plore®8000	AR HE-F001 (P3 rated**)	100
CleanSpace	CleanSpace 2	PAF-0037 (P3 rated**)	100

*Allows a 1.0% particle penetration

**Allows a 0.05% particle penetration

**Table 3:** Various petitions by US underground coal mine operators to use non-approved PAPRs

Docket	Year	Petitioner	Regulation	PAPR	Ref.
M-2020-015-C M-2020-016-C	2020	Westmoreland San Juan Mining LLC, San Juan Mine 1 MSHA I.D. No. 29–02170 Waterflow, NM	75.500(d) 75.507–1 75.1002	TR–800 PAF–0060	[35, 36]
M-2019-067-C M-2019-068-C M-2019-069-C	2021	Peabody Twentymile Coal Mining Foidel Creek Mine Routt County, CO	75.500(d)	TR-800 EX	[37–39]
M–2021–007–C M–2021–008–C	2021	Mountain Coal Co. LLC West Elk Mine MSHA ID No. 05–03672 Somerset, CO	75.507–1(a)	TR–800	[40, 41]
M-2021-010-C M-2021-011-C M-2021-012-C	2021	Consol Pennsylvania Coal Co. LLC, Bailey Mine MSHA ID No. 36-07230 Greene County, PA	75.507-1(a) 75.500(d) 75.1002(a)	TR-800 EX	[42–44]
M-2021-013-C M-2021-014-C M-2021-015-C	2021	Consol Pennsylvania Coal Co. LLC, Harvey Mine MSHA ID No. 36-10045 Greene County, PA	75.507–1(a) 75.500(d) 75.1002(a)	TR-800 EX	[45–47]
M-2021-016-C M-2021-017-C	2021	Consol Pennsylvania Coal Co. LLC, Enlow Fork Mine MSHA ID No. 36-07416 Washington County, PA	75.507–1(a) 75.500(d) 75.1002(a)	TR-800 EX	[48, 49]
M-2021-045-C	2022	Signal Peak Energy, LLC Bull Mountains Mine No. 1 I.D. No. 24-01950	75.1002(a)	TR-800	[50]

The main concern when implementing PAPRs in coal mining is the requirement of intrinsically safe devices, which is not necessary in some other types of mines. While the first PAPRs were implemented in the 1970s in coal mines, and many improvements have been made since those early models, they still require MSHA approval before implementation [33]. Table 3 shows some coal mining companies that have petitioned for the implementation of PAPRs. These petitions refer to specific PAPR models that are already approved and meet the required standards. The TR-800 is manufactured by 3M, and the PAS-0060 and EX are manufactured by CleanSpace. All of these PAPRs are also intrinsically safe.

### Healthcare Industry

Healthcare workers are exposed to several microorganisms that may cause diseases like influenza and others. To lower the risk of infection, respiratory protection equipment is required [51]. As an example, with the emergence of severe acute respiratory syndrome (SARS) disease in 2003, Singapore implemented strict infection control measures where, apart from other personal protective equipment, the use of N95 masks was implemented. PAPRs were mandatory for high-risk or aerosol-generating procedures. A study on 51 healthcare workers showed that despite some communication or visual limitations, the use of PAPRs in the SARS context is strongly recommended. This is because SARS is considered highly contagious and more deadly than tuberculosis [52]. A review paper that summarizes different publications on the use of PAPRs against highly infectious diseases reported many inconsistencies between the studies done in this area. However, it found that, compared to normal APRs, workers using PAPR observed much lower contamination on the clothes and skin. Workers also reported an increase in their comfort during work [53].

The recommendations on the precise level of respiratory protection for aerosol-generating procedures vary across international governing bodies. However, they generally recommend different respirators including N95, FFP2, or FFP3 masks [54]. Results from the comparison of these respirators, also including a P100 filter like the ones commonly found in PAPRs, are shown in Table 4 [55]. The N95 standard refers to a 95% efficiency when non-oil particles are present. FFP2 and FFP3 are classifications according to the European EN 149 standard, with efficiencies of 94% and 99%, respectively [56]. The P100 has an efficiency of 99.97% and is resistant to oil particles [57].

**Table 4:** Filtration efficiency comparison

Respirator class	N95		FFP2		FFP3		P100	
Manufacturer	M1	M2	M1	M2	M1	M2	M1	M2
Mean penetration (%)	0.703	0.565	0.270	0.505	0.0098	0.0144	0.0034	0.0222
Standard deviation	0.200	0.525	0.096	0.275	0.004	0.011	0.002	0.036

### Other Industries

In the automobile industry, FFP2 and FFP3 certified negative-pressure half masks were studied for their protection against diesel exhaust particles. The average filtration efficiencies were observed to be in the 80–86% range for the two tested masks. The study also showed that the most penetrating particles were in the range of 30–300 nm [58]. During the 1970s, PAPRs were introduced to the UK coke industry, lowering the exposure of the coke oven workers to polycyclic aromatic hydrocarbons. Their prolonged inhalation can cause lung cancer [59]. Studies focused on the lead industry showed that PAPRs had a mean effective protection factor of 18.2. This is lower than the assigned protection factor of 50 [60]. In 1983, a study during silica bagging operations suggested that the respiratory protection of some PAPRs was lower than the expected APF with particle mean size between 5.8 and 7.1 $\mu m$. However, the protection factors for PAPRs with full-face masks were between 25 and 215, and for PAPRs with half-face masks between 16 and 193 which was still higher than for other types of masks [61]. These reports show the successful implementation of PAPRs and related masks across several industries.

## PAPR and Industry Exposure Standards

Predominantly, three agencies in the USA work to regulate the workers’ workplace exposure. They are the National Institute for Occupational Safety and Health (NIOSH), the Occupational Health and Safety Administration (OSHA), and the Food and Drug Administration (FDA). NIOSH conducts research and makes recommendations to prevent work-related injuries and diseases, including surface and underground mining operations. It is responsible for the testing and approval of any new respirator under the requirements defined in Title 42, Part 84 of the Code of Federal Regulations (42 CFR 84) [62, 63]. NIOSH researches and develops standards and user guidance. OSHA comes into play when a respirator is needed in a general industrial workplace. It establishes the respiratory protection standard, which mentions that NIOSH must approve any respirator, and employees must pass medical evaluations, fit testing, and training. Finally, the FDA provides the marketing clearance for the respirators. It also provides further review when the respirators are intended to be used as a protection against a specific disease or infection [64]. As the use of PAPRs increases, new models continue to emerge. However, most models could fail to meet the safety and protection requirements in certain regions due to the lack of unified standards. Therefore, different countries started to implement standards (see Table 5) for PAPRs [65].

**Table 5:** PAPR standards around the world

Country	Standard
USA	NIOSH 42 CFR 84. Section K PAPR
	ANSI AIHA Z88.7-2010
Japan	JIS T 8153:2002
	JIS T 8154:2018
	JIS T 8157:2018
UK	BS EN 12941:1998+A2:2008
	BS EN 12942:1998+A2:2008
China	GB 30864-2014
	GB/T 23465-2009
Other standards	ISO/TS 16976-1:2007

It is also important that the PAPRs can keep concentrations under the permissible exposure limits (PEL) for each type of dust particulate. This reinforces the importance of WPF, as this will indicate the protection level against the specific type of dust in the workplace. The mandated permissible concentrations of some unique types of dust particles are listed in Table 6. The time-weighted average (TWA) is the maximum average concentration in 8 h and is calculated using Eq. 2, where $C_i$ is the concentration, $T_i$ is the exposure time for that concentration, and it is divided by 8 h, no matter the real exposure time. Short-time exposure is the maximum exposure in 15 min. Concentrations can be calculated in parts per million (ppm), parts per billion (ppb), or mass per unit volume.

2 $$\begin{aligned} TWA=\frac{\sum C_iT_i}{8} \end{aligned}$$

**Table 6:** Some exposure standards

Chemical	Standards	Comments	Reference
Coal dust	1.5 mg/m^3^	Respirable dust underground	[66]
	0.5 mg/m^3^	Concentration in intake air	
Aluminum metal	15.0 mg/m^3^	Total dust	[67]
	5.0 mg/m^3^	Respirable fraction	
Asbestos	100,000 fiber/m^3^	Collected in a 400 L sample over 100 mins (method # 7400)	[68]
Calcium Carbonate	15.0 mg/m^3^	Total dust	[67]
	5.0 mg/m^3^	Respirable fraction	
Calcium silicate	15.0 mg/m^3^	Total dust	[67]
	5.0 mg/m^3^	Respirable fraction	
Limestone	15.0 mg/m^3^	Total dust	[67]
	5.0 mg/m^3^	Respirable fraction	
Silica (crystalline) Respirable quartz	$\frac{10 mg/m^{3}}{(\%SiO_2)+2}$	Depends on the fraction passing a size selector with specific characteristics	[69]
Wood	3.0 mg/m^3^	Hardwood	[70]
	5.0 mg/m^3^	Softwood	
N-Methylpyrrolidone (NMP)	82 mg/m^3^	Maximum allowable concentration	[71]
CO	9 ppm	TWA	[72]
Lead	0.15 $\mu$g/m^3^	3-month average	
NO_2_	53 ppb	Annual mean	
O_3_	0.07 ppm	TWA	
SO_2_	0.5 ppm	3-h exposure	

## Performance Evaluation of PAPRs

The respiratory protection level (respirator efficiency) of PAPRs depends on several factors. These include breathing airflow, blower airflow, user efforts, and leakages. Although not quantifiable easily, the worker’s comfort and communication ability are also essential for PAPR evaluation. The following subsections compare the PAPRs with other respirators and describe their workplace performance, comfort, and other factors.

### Performance Testing for Fit Factors

PAPRs have been compared with other types of respirators to assess the differences in protection levels. A study at the Jewish General Hospital in Quebec, Canada, compared a PAPR with an N95 mask. The research showed that despite having a higher filtration efficiency, using PAPRs presented communication difficulties. The power requirement for the blower also impedes nominal operations. The users could not efficiently use equipment such as a stethoscope [73]. Surveys conducted in Singapore during the SARS pandemic showed that 84% of healthcare workers prefer using PAPRs instead of N95 masks [74].

**Table 7:** Leakage ($\%$) while using respirators under different wearing conditions [75]

Wearing method	RPR	PAPR
Recommended	1.82	0.18
Knit	10.92	0.23
Towel	6.39	0.42
Helmet	3.19	0.23

Like any personal protective devices (PPEs), the PAPRs must be used according to the recommendations for best performance. For example, PAPR’s efficacy in protecting a user from respirable dust depends strongly on its proper fit. There must be an adequate seal between the user’s face and the respirator to eliminate any leakage resulting in particulate transportation within the respirator. Two studies compared the Koken Sakai-Type 1180C-05 replaceable particulate respirator (RPR) and the Koken BL-321S PAPR. Table 7 shows the leakages for the respirators against the wearing conditions. The particles measured were atmospheric dust with a particle size of at least 0.5 $\mu$m. Leakage rates for RPRs range between 1.82 and 10.92%, while for PAPRs range between 0.18 and 0.42%. In this table, the “recommended” wearing method indicates the case where all the manufacturer’s guidelines were followed to achieve optimum protection. However, workers sometimes place a knit or a towel between the mask and their faces. At other times, the workers use a helmet and place the straps over it, stretching them and changing the fitting of the mask. Table 8 shows the fit factors (FF) for an RPR and PAPR for these wearing methods and different efforts by the users [75, 76]. Finally, the protection with a PAPR and an N95 mask arranged in series was evaluated under different airflow conditions, including a PAPR failure scenario [77]. Some of the results are shown in Table 9. Even though the results show high protection factors, the paper does not mention pressure drop or changes in breathing difficulty.

A commentary published by Nicas mentions some possible errors that may lead to overestimating the APF reported by OSHA in half-face masks [78]. This issue may easily apply to PAPRs, too. He mentions that different factors such as sampling errors, particle characteristics, or leakage were not considered while establishing the protection factor for the respirators. These errors may lead to reporting a higher APF. One of the principal factors that may contribute to the error is the particle deposition in the respiratory tract during the inhalation and exhalation cycle. This deposition should be corrected when calculating the APF, as it affects the sampling of the particles.

**Table 8:** Fit factors for an RPR and PAPR under different work rates and wearing conditions [76]

Respirators	RPR		PAPR	
Wearing conditions	Rest	Exercise	Rest	Exercise
Recommended	145.3	84.8	786.5	444.5
Knit	12.3	9.3	762.4	396.6
Helmet	118.7	68.2	737.2	468.9

### Performance Testing for Physical Effort

Another widely tested parameter that determines the PAPR efficacy is the influence of the physical effort by the user. The user effort is usually quantified in terms of the variable oxygen flow (VO_2_) rate. This is important as the PAPR should be capable of maintaining positive pressure to avoid leakages for all efforts. However, if the wearer’s breathing airflow requirement is higher than what is supplied by the blower, a negative pressure is created. This causes contaminants to leak into the mask. This condition is referred to as “over-breathing.” The interaction between the work rate and airflow has a significant impact on the FF. This has been shown through several measurements during tests on manikins [24]. In a study that used an automated breathing metabolic simulator, negative pressures were detected at an average oxygen consumption of 3.0 L/min [79]. Breathing airflow of persons exercising at 80–85% of their maximum VO_2_ can reach peaks of up to 400 L/min. It is important to explain that while oxygen consumption refers to the amount of oxygen a person consumes, the breathing airflow is much higher, as it refers to all the components of the air and not just the oxygen. This is higher than the minimum 170 L/min required for loose-fitting PAPRs and 115 L/min for tight-fitting PAPRs. The study also mentions that the battery charge can greatly influence the performance of the blower, increasing the over-breath volume as the charge decreases [80, 81]. Another study showed that there is an influence of the airflow on the leakage into the respirator. For the study, a hood and a blouse were used; the results for the hood, as this is the most common one, are shown in Table 10. These are two types of loose-fitting PAPRs. The principal difference is the space inside the respirator, known as the dead space. The amount of air available in this space will impact the leakage when more air than provided is needed.

**Table 9:** APF at different breathing air flows and with different mask combinations [77]

Airflow (L/min)	Combination	APF
25	PAPR	31,552
	N95	143
	PAPR + N95	325,037
	PAPR Failure	8
	PAPR Failure + N95	1990
40	PAPR	33,741
	N95	98
	PAPR + N95	320,185
	PAPR Failure	9
	PAPR Failure + N95	1414

**Table 10:** Hood leakage and CO_2_ concentration at different flow rates [82]

Flow rate (L/min)	Leakage (%)	CO_2_ concentration (%)
180	0.010	0.25
180	0.020	0.35
120	0.020	0.45
90	0.045	0.55
60	0.290	0.65

**Fig. 2:**
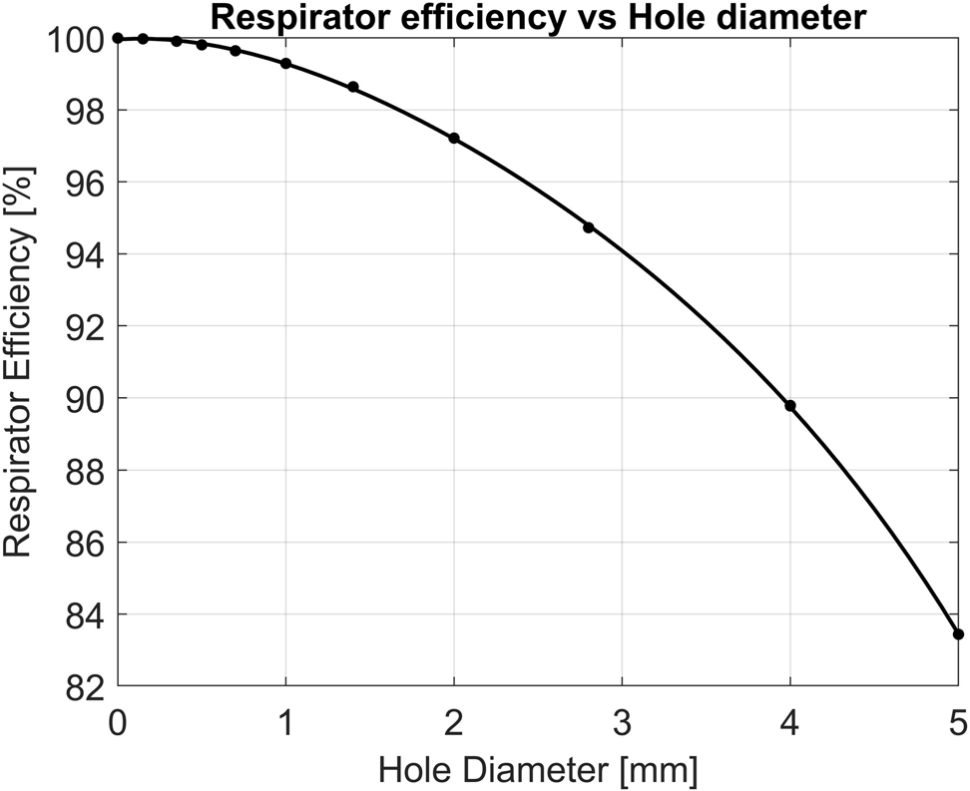
Hole diameter effect on PAPR performance [83]

The PAPR fitting also plays an important role in controlling leakage. A manikin-based study used two improperly sized loose-fitting masks and a stretched-out mask to measure the impact this may have on the protection factor. Results showed that the manikin fit factor (mFF) decreases exponentially as the mean inspiratory flow (MIF) increases. This follows previous studies on the influence of over-breathing. Research has shown that improperly sized masks still provide acceptable protection up to a moderate workload. However, the stretched-out mask did not reach the minimum APF of 25, not even at low workloads [25]. Another study showed the performance of a tight-fitting mask when the face seal is compromised [83]. They used different diameter holes to study the capability of the mask to compensate for the leakage through the holes. Results showed diminishing respirator efficiency. This is shown graphically in Fig. 2. The figure compares the efficiency as the hole diameter increases, simulating leakage rates increase. As expected, a higher leakage means a lower efficiency of the respirator.

### Workplace Protection Factor

The protection factor has also been tested under real or simulated work conditions. The factor obtained through this process is referred to as the “workplace protection factor” (WPF). However, neither NIOSH nor the manufacturers recommend a field test method [84]. There are very few studies on the WPF determination of a respirator. For example, two studies measured the WPF during aircraft sanding and painting operations [19, 85]. The WPF was measured for each operation and for different elements. Table 11 summarizes the results obtained during these studies. For the sanding operations, the samples did not reach the minimum concentration required to measure the protection factor scientifically. In a Japanese study that focused on a few occupations that lead to workers’ exposure, three respirators, including a PAPR, were compared using the WPF. Research results showed that the PAPR showed WPFs between 16 and 993, with an average being 117 [18].

**Table 11:** Aircraft sanding and painting WPFs

Operation/element	Protection factor
Painting	$> 1000$
Sanding	*NA*
Strontium	$> 54,000$
Magnesium	$> 20,500$

The healthcare industry uses PAPRs significantly due to the nature of exposure of medical professionals. In research in this setting, a simulated workplace protection factor (SWPF) was evaluated before, during, and after chest compression for the HALO CleanSpace PAPR model. This enabled the understanding of WPF changes with work intensity [16]. The average values for the SWPF were 3576 before the compression, 4290 during the compression, and 4135 after the compression. The principal limitation of the study was the small number of participants. However, this research showed that the SWPF does not change significantly with the extent of work intensity. A larger study with 144 measurements per device and 11 devices showed that the protection factor can exceed 250,000 [17].

### Comfort and Other Parameters

An important parameter to consider when using a PAPR is the wearer’s comfort, especially if the device has to be used for long periods. A common concern can be the effect on the wearer’s mobility. One research project studied the user’s comfort in a confined space (ambulance). It was shown that the time taken by a user, with a PAPR, to complete four tasks was 3–5 s longer when compared with traditional equipment. However, when the participants rated the ease of breathing on a 1–5 scale, the PAPR obtained almost three more points (favorable) than a regular air purifying respirator [74]. It is also critical to note that breathing releases a higher concentration of CO_2_ into the PAPR filter. Due to unique filter characteristics, this leads to different concentrations inside the respirator filter as shown in Table 12. The CO_2_ concentration in the PAPR is much lower compared to other masks or respirators.

**Table 12:** CO_2_ concentration (%) inside different types of masks [86]

Face mask	CO_2_ Concentration
No mask	0.26
PAPR	0.59
KN95	2.60
Valved respirator	2.40

Ease of communication is essential in many tasks. The PAPR’s blower and the mask can affect the communication ability of the wearers. The noise produced due to PAPR blower operations can reach 56 dB; this affects communication significantly [87]. A study using loose-fitting masks showed that word discrimination decreases to 48% at 45 db HL with an NU-6 word list test. It showed no difference between having the auricle inside and outside of the mask [88]. The HALO CleanSpace was also tested for speech recognition under three parameters: a rhyme test that requires the subjects to listen and select words from a list of similar-sounding words; a medical phrases word understanding test, where the subjects had to listen and write medical phrases; and a medical phrases meaning test, where the amount of keywords listened to correctly was recorded. The use of a headset was also implemented as an option to improve communication. Results are shown in Table 13. Therefore, good communication is achievable with the help of a headset, reaching levels similar to the ones achieved by using a surgical mask [16].

It is also important to note that many tasks rely on the olfactory system to help detect contaminants that may be a risk to the workers’ health. One of the best examples is natural gas, which does not have a smell. However, companies add a harmless chemical called ethyl mercaptan, C$_2$H$_5$SH, to give it its distinctive odor so that people can detect any leaks swiftly [89]. When using a PAPR or an N95 mask, the sensitivity to odors is reduced. A study concluded that even though using a PAPR can affect sensitivity, it does not affect odor discrimination [90]. Further related studies were suggested by the authors. This could be challenging for the miners, especially if mines use a stench gas system for emergency alerts.

**Table 13:** Ratings (%) from communication tests for the HALO CleanSpace [16]

Test	Without headset	With headset
Rhyme test	70	84
Medical phrases word recognition	98	97
Medical phrases meaning understanding	95	95

The use of a PAPR can cause different physiological effects on the wearer due to inhalation of gases, pressure, and temperature changes. As shown by previous studies, the work rate greatly impacts the PAPR performance and the wearer’s comfort. Even though all of these parameters may have an impact on the wearer, the temperature difference between ambient and inhaled air temperature may not be felt by the wearer, as the minimum temperature change that men can feel is 0.94 $^{\circ }$C. Women, in general, are slightly more sensitive to temperature changes and can detect changes of 0.80 $^{\circ }$C. The maximum temperature difference was 0.90 $^{\circ }$C at rest and low energy expenditures [91, 92].

## CFD Modeling for PAPR Performance Prediction

Computational fluid dynamics (CFD) is an efficient fluid flow modeling and prediction tool. It works by approximating the solution to Navier-Stokes equations through the discretization of a demarcated flow volume. It has been used to study the efficiency of different filtration media and to uncover the performance mechanism of the filter elements. CFD models also facilitate the understanding of the behavior of particles in a closed environment [93, 94]. An underground mine is one such confined environment where CFD modeling can play a crucial role in improving worker protection. It can simulate different conditions, including dangerous gas and particle concentrations, and enable the creation of better risk management plans to ensure workers’ safety. In the context of PAPRs, it can help to understand the advantages and challenges associated with their implementation. Therefore, CFD modeling studies can provide insights into their performance under unique conditions.

**Fig. 3:**
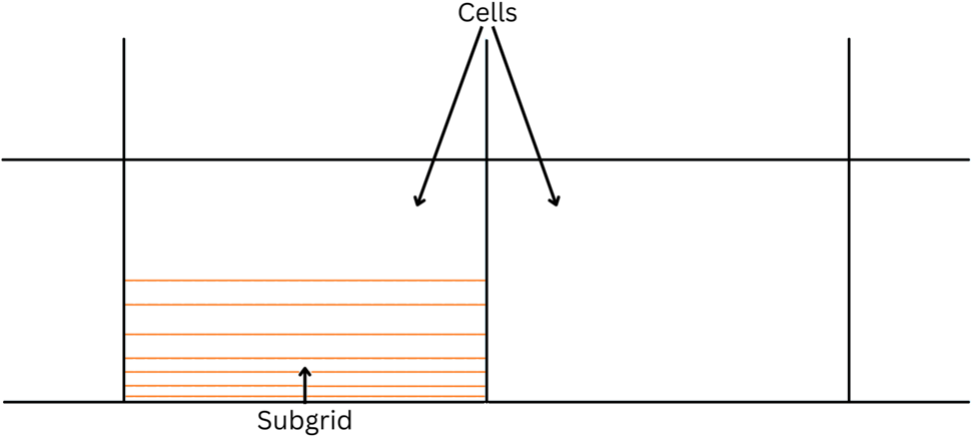
1D subgrid [99]

The biggest problem when trying to simulate models where small details are important (e.g., filters) is the computational power required. The most general approach to reduce the computational power required is to increase the size of the mesh elements. However, this may result in a decrease in the model’s accuracy. When using a coarse mesh, particles are grouped into large clusters; scaling is applied to obtain the dominant forces and predict the particles’ behavior. This may result in a lack of accuracy, as forces are not calculated directly from the particles [95]. This is not useful when trying to study filtration media, as particles cannot be treated as a group. As any filtration media needs a very fine mesh to capture every detail, the computational cost can increase rapidly. Therefore, the subgrid approach is often used [96]. It is important to mention that subgrid models can also be used with turbulence models, large eddy simulations (LES), and even with more complex models such as the energy-minimization multi-scale (EMMS) [97, 98]. Myers and Walters show an example of a one-dimensional subgrid [99]. It uses a set of differential equations to solve for the parameters used to calculate the primary grid solution. The subgrid is shown in Fig. 3.

### Porous Media Flow Modeling

Several techniques are used to model flow through porous media, including CFD-DEM coupling. This technique uses the discrete element method to model the particle’s physical interactions. It, then, couples CFD to solve the fluid flow [100]. However, this can be very expensive in terms of the computational power required, as the mesh can be too fine or complicated. For example, research has identified unique challenges while using different approaches to porous media modeling. One such research focused on oil filtration. For laminar incompressible flows, this research suggested using the Navier-Stokes-Brinkman model, which includes the equations of time-dependent momentum balance (Eq. 3a), continuity (Eq. 3b), and transport (Eq. 3c) as shown below [96, 101].

3a $$\begin{aligned} \frac{\partial \overrightarrow{u}}{\partial t}-\nabla \left( \widetilde{\mu }\nabla \overrightarrow{u} \right) +\left( \rho \overrightarrow{u},\nabla \right) \overrightarrow{u}+\widetilde{\mu } K^{-1}\overrightarrow{u}+\nabla p=\overrightarrow{f} \end{aligned}$$

3b $$\begin{aligned} \nabla \overrightarrow{u}=0 \end{aligned}$$

3c $$\begin{aligned} \frac{\partial C}{\partial t}+\left( \overrightarrow{u},\nabla C \right) =D\Delta C-\alpha C \end{aligned}$$

where $\overrightarrow{u}$ is the velocity, *p* is the pressure, *C* is the particles’ concentration, $\widetilde{\mu }$ is the viscosity, *K* is the filter media permeability, *t* represents time, and $\rho$ is the fluid density. The diffusion coefficient and the absorption rate for the porous media are represented by *D* and $\alpha$, respectively. The symbols $\Delta$ correspond to change and $\nabla$ to the spatial gradient.

### Filter Simulation

**Fig. 4:**
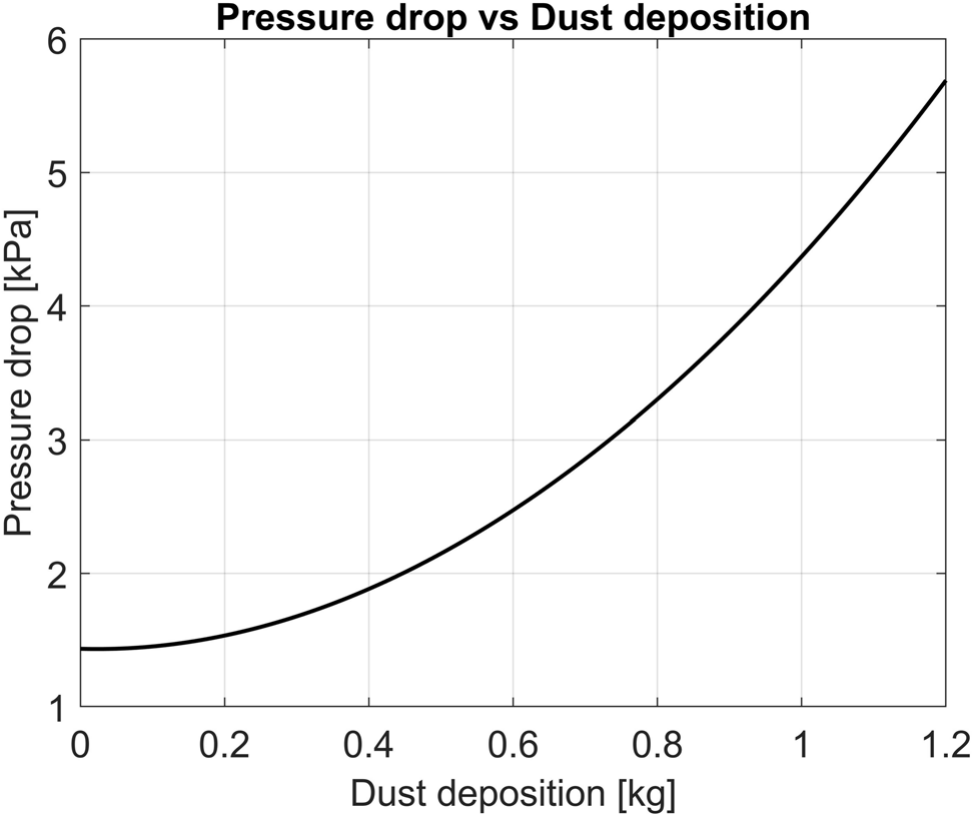
Pressure drop as dust is accumulated [103]

**Fig. 5:**
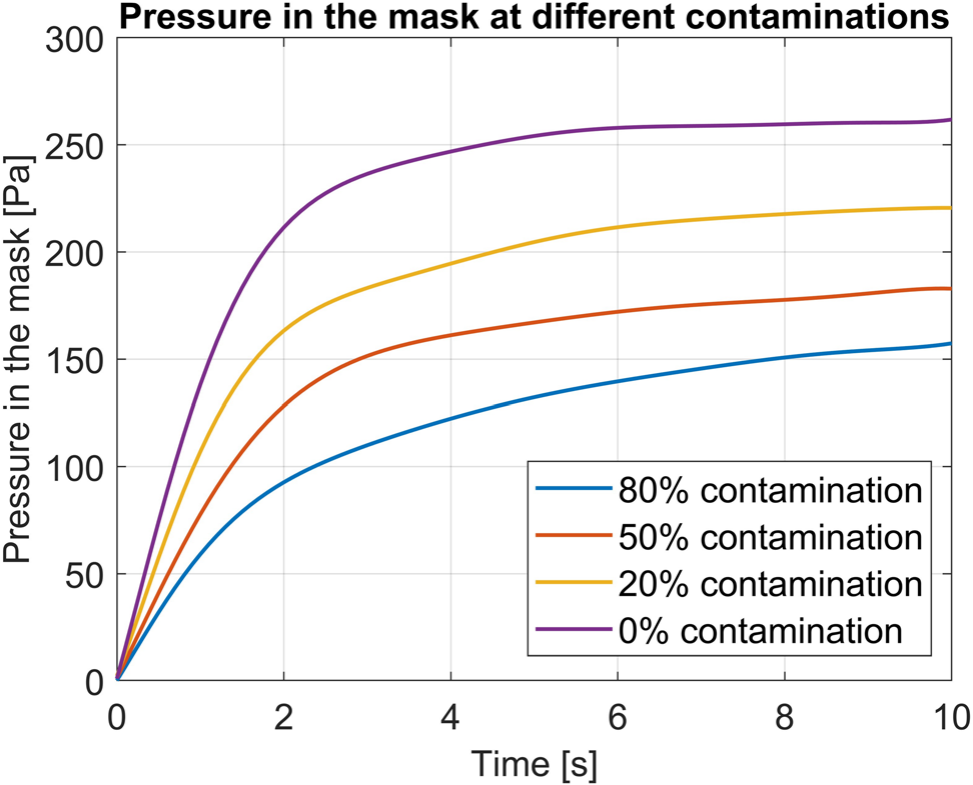
Pressures inside the mask at different filter contamination [102]

HEPA filters are used as the primary filtration elements in the PAPRs. They have a pleated design and can remove up to 99.97% of particles of sizes 0.3 $\mu m$ and larger. A rapid increase in the pressure drop as the particle build-up occurs is the major drawback of these filters (Fig. 4). This severely limits the useful operation time in the short run (a shift, for example). The life of the respirator is also affected on a longer time scale if it is not maintained periodically. Pressure changes within the mask have been studied by several researchers. The authors of this article extracted the raw data model from models developed by Cheberiachko et al. using ImageJ software [102]. Plots of the best-fit polynomials for those discrete points are shown in Fig. 5. Clearly, the pressure within the mask progressively diminishes with the particle accumulation.

**Fig. 6:**
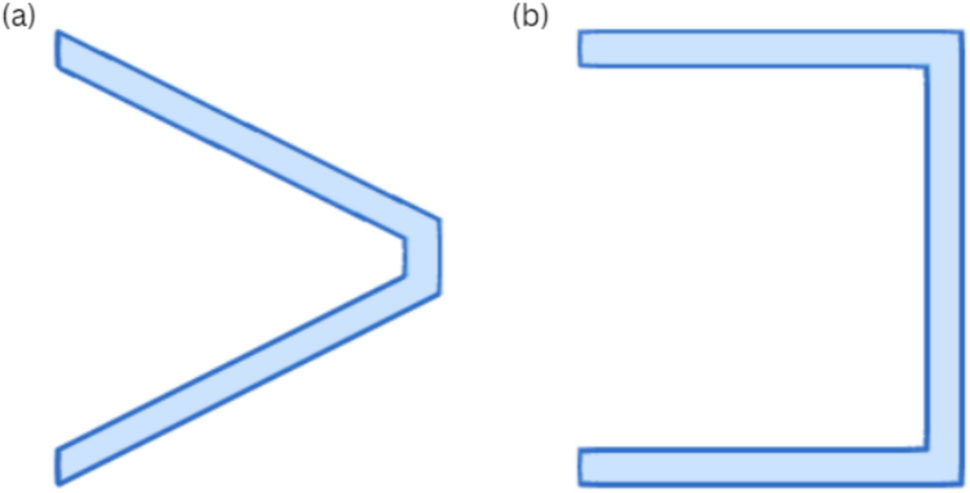
Pleated filters’ structures: **a** trapezoidal, **b** rectangular

Dust deposition can be modeled using the Lagrangian particle tracking method, which can be implemented in open-source or commercial software. These models assume that the dominant force acting on a particle is the air drag. When the particle Reynold’s number (*Re*_p_) condition in Eq. 4a, with inlet velocity $u_{in}$, $d_p$ as the particle’s diameter, and air density $\rho$ is met, the governing equation is Eq. 4b, where $v_{ip}$ is the particle velocity, *t* is the time, $\mu$ is the viscosity, and $\rho _p$ is the particle’s density. $C_c$ represents the Stokes-Cunningham slip correction given by Eq. 4c, where $\lambda$ is the molecular mean free path, *d* is the pleat distance, and $v_i$ is the relative fluid’s velocity [104, 105]. Figure 6 shows the (i) trapezoidal and (ii) rectangular structures of pleated filters [106].

4a $$\begin{aligned} Re_p=(\rho u_{in} d_p/\mu )<1 \end{aligned}$$

4b $$\begin{aligned} \frac{dv_{ip}}{dt}=\frac{18\mu }{d_{p}^{2}\rho _pC_c}(v_i-v_{ip}) \end{aligned}$$

4c $$\begin{aligned} C_c=1+\frac{2 \lambda }{d}(1.257+0.4e^{-1.1d/2\lambda }) \end{aligned}$$

**Fig. 7:**
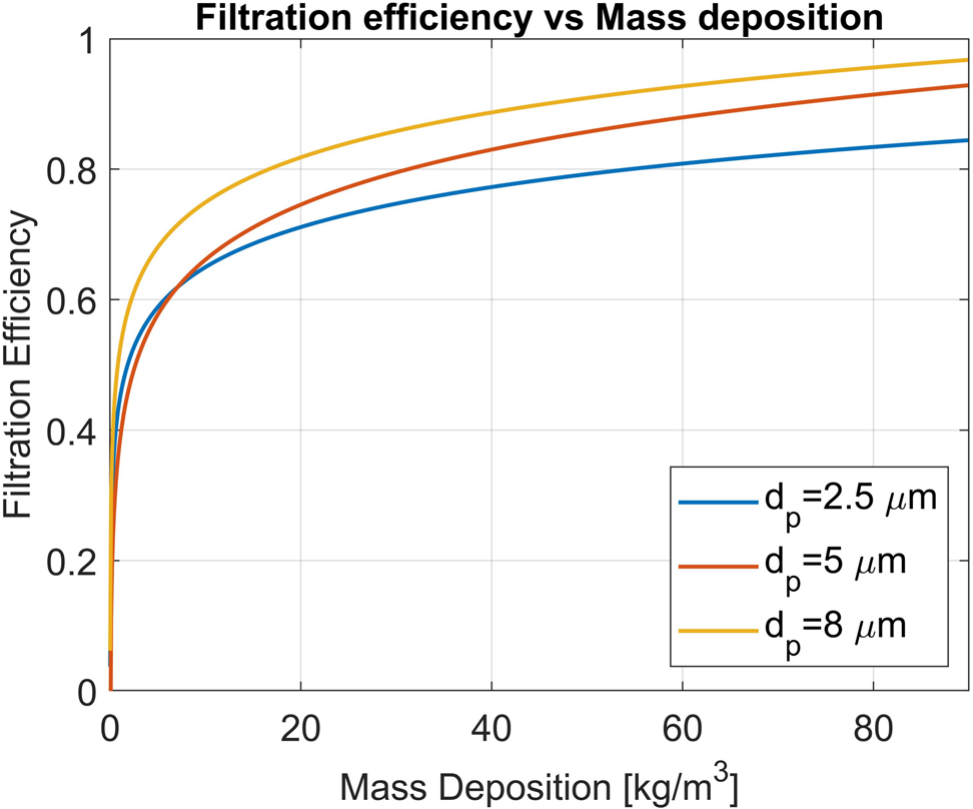
Filtration efficiency for different particle sizes with a fiber diameter of 15 $\mu$m and an SVF of 19%[108]

Different models for pleated filters have been evaluated for their accuracy. Comparison with the experimental data shows that the Reynolds stress equation model (RSM) is more accurate when predicting the pressure drop across the filter under turbulent flow regimes than the general $\kappa$-$\epsilon$ models. All of these are turbulence models with second-order upwind discretization schemes. Table 14 shows the comparison between the simulated and measured data [107]. The CFD-DEM modeling approach uses the discrete element method to model the behavior of the particles and a CFD model to simulate the fluid behavior. When both models are coupled, interactions between the particles and the fluid can be simulated. This approach can give information such as interaction forces, collision forces, fluid forces (e.g., buoyancy), and adhesion forces. Yue et al. researched the filtration process using this approach and calculated the filtration efficiency [108]. The results are shown in Fig. 7. The efficiency was observed to improve with an increase in the aerosol diameter.

**Table 14:** RSM and experimental pressure drop comparison

Air velocity (m/s)	Experimental (Pa)	RSM (Pa)
0.5	37	30
1.0	81	71
1.5	123	123
2.0	179	185

### Modeling PAPRs

**Fig. 8:**
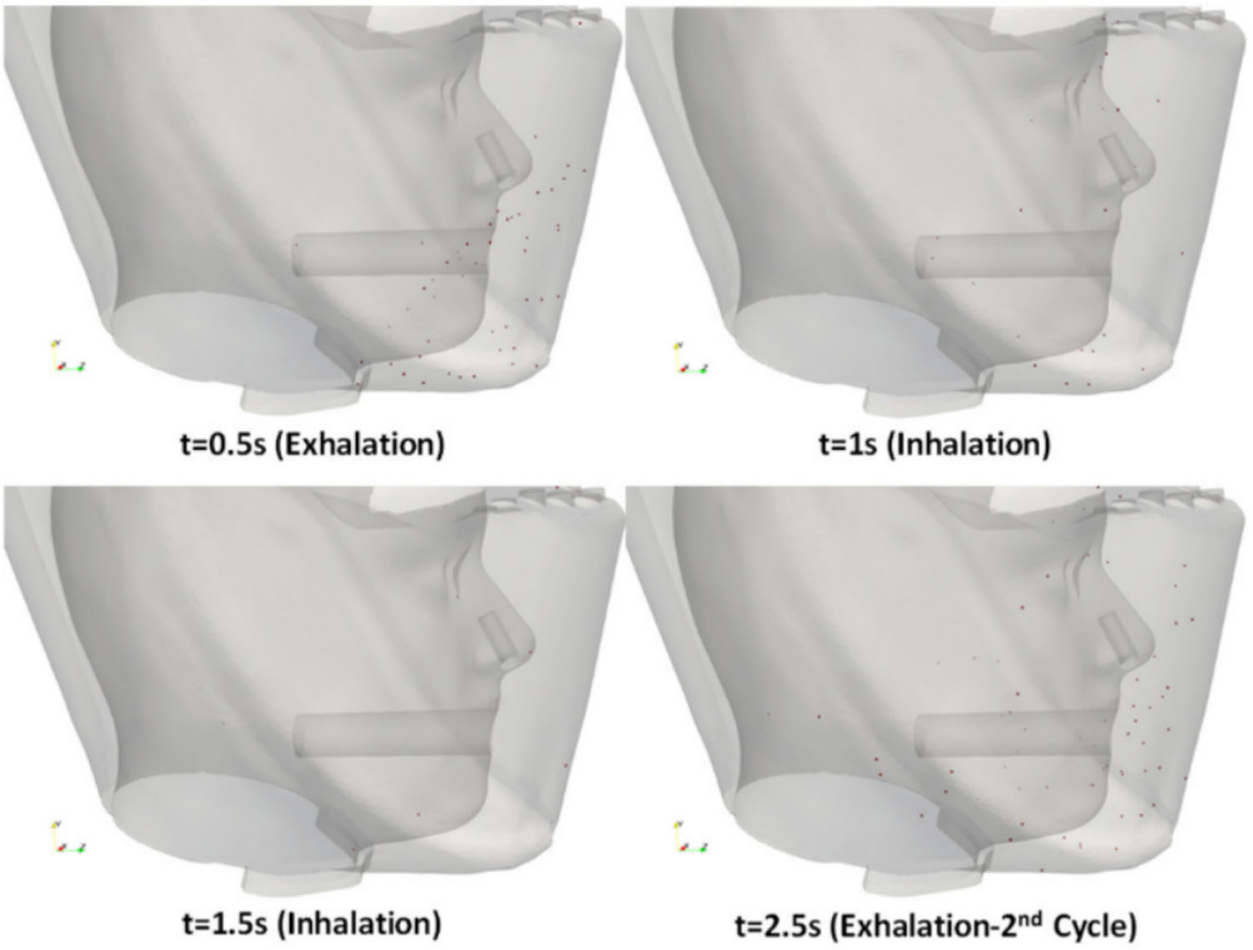
Distribution of particles during a breathing cycle [109]

There are not many studies on the development of CFD models for the PAPRs. For example, Xu et al. studied the flow of exhaled particles inside the PAPR. The study used the OpenFOAM software to track the particle distribution during a breathing cycle; this is shown in Fig. 8. This study gives a clear idea of the behavior of particles that were able to pass the filters and the number of them that reached the wearer. It also shows that leakage increases as the particle size decreases [109]. Studies of respirator fit and leakages, critical factors for efficiency, can be carried out using these models. When studying leakage, it is important to consider the path that the particles will follow inside the mask to determine whether they are reaching the user [110].

**Fig. 9:**
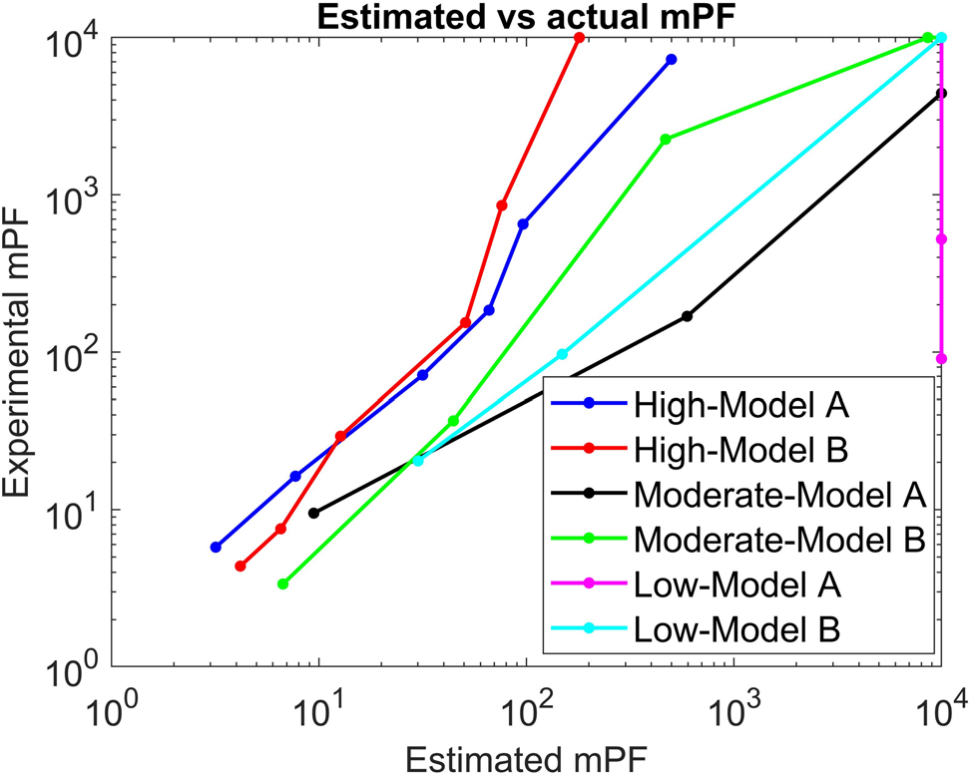
Comparison between experimental mPF and CFD predicted mPF [111]

Another study developed CFD models to predict the manikin protection factor (mPF) of loose-fitting PAPR and then compared them to experimental values. The results showed the models’ ability to accurately predict the protection factor of the respirators [111]. With the software ImageJ, an approximation of the results was obtained and is shown in Fig. 9. This figure compares the real protection factor and the estimation obtained through simulations. The study used two different PAPRs and three different work rates.

## Innovation and other Current PAPR Research

As explained in the previous sections, PAPRs are efficient personal scrubbers that can significantly lower the exposure of users to harmful aerosols. Current research is focused on solving different problems, such as the build-up of some CO_2_ concentration in the mask’s dead space, that PAPRs may have. Minimizing or eliminating leakages while mimicking human breathing patterns is also being studied extensively. Suitable solutions to these problems will encourage the adoption of PAPRs widely.

### Independent Breathing Simulation Studies

Breathing patterns are one of the most important factors that may affect the results of any PAPR study. Factors such as airflow, work rate, and sudden changes in the pattern need to be addressed to replicate real human respiration. When simulating breathing patterns, sine, triangle, and square waves are generally used. This, however, is still far from a real pattern. A study proposed a simulator capable of producing the three patterns mentioned before, but it could also generate random patterns from a PC or recorded data. It could also duplicate a breathing pattern in real time. The aim was to be able to test respirators under realistic conditions [112].

Another study recorded different parameters during the cleaning of an “Isolation Unit.” The objective of the study was to define the minimum operational flow for loose-fitting PAPRs, based on the breathing parameters of the wearers. Data gathered included the minute volume (MV), mean inhalation flow (MIF), and peak inhalation flow (PIF). The results of this study are shown in Table 15 [113]. This table shows the mean and maximum values for each parameter. As can be seen, even the maximum values are lower than the minimum requirement of 170 L/min established by NIOSH. However, these values can be higher as the work rate increases.

**Table 15:** Breathing parameters

Parameter	Mean	Maximum
MV (L/min)	33	41
MIF (L/min)	74	97
PIF (L/min)	107	107

**Fig. 10:**
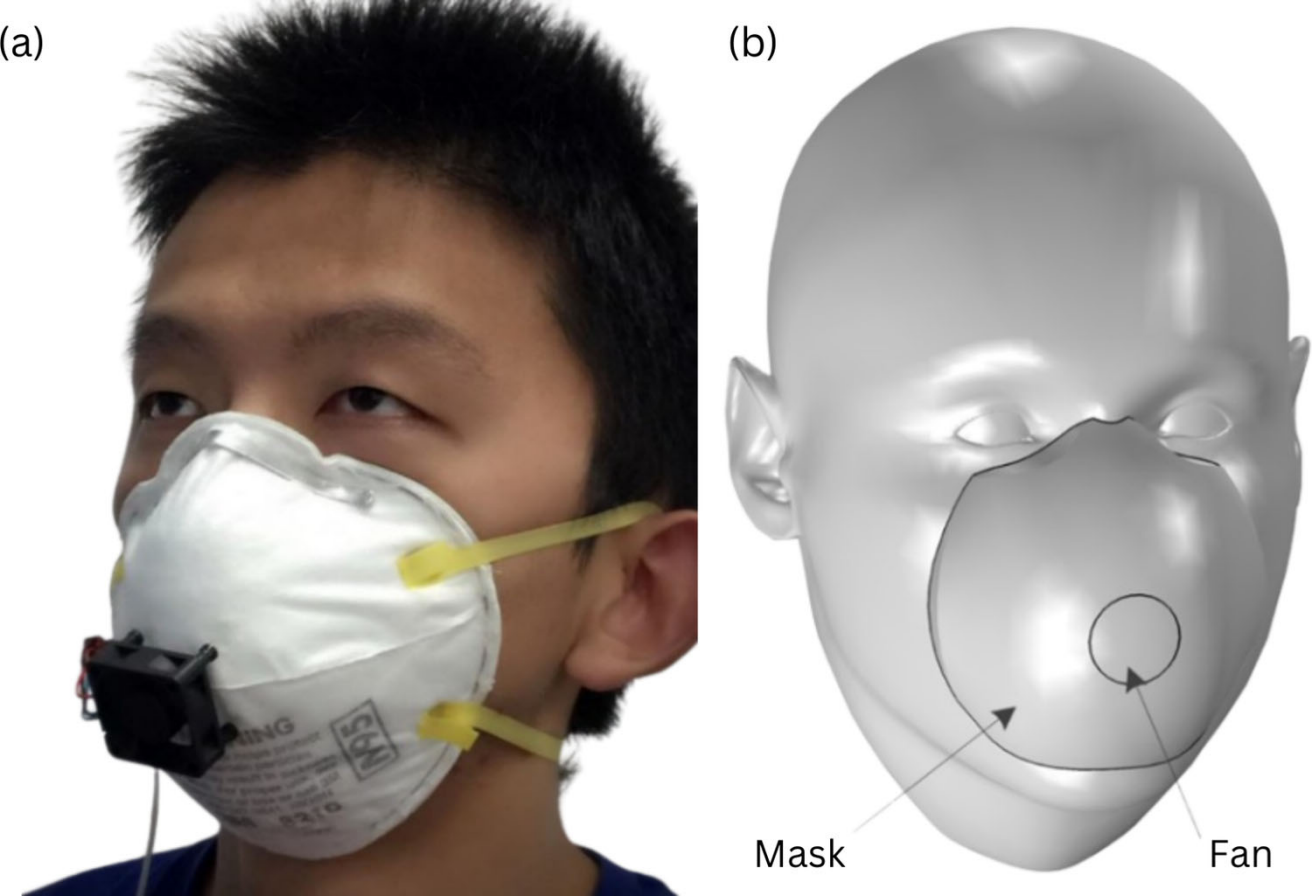
Experimental models for CO_2_ reduction [114, 115]

### Realistic Breathing under External Airflow

There are a few studies that have integrated realistic breathing through masks under known ambient airflows. Two studies added a fan directly in front of an N95 mask. The goal of this arrangement was to lower the CO_2_ concentration. The first study showed that exhaled air can contain up to 5.0% of CO_2_ compared to the average of 0.03% found in ambient air. The model added a fan in front of the mask as shown in Fig. 10a. The experiments showed a decrease in the CO_2_ concentration to 2.0% and a decrease of 2 K in the temperature. Computer simulations showed a reduction of CO_2_ concentration to 0.3% when a fan was placed across the filter, as shown in Fig. 10b. However, this may have compromised the efficiency of the mask [114, 115]. As none of these studies measured the APF of the experimental models through experiments or simulations, further research should be performed.

A study was recently performed on the feasibility of an ultra-portable low-cost PAPR in response to the high demand for the equipment during the SARS-COV-2 pandemic. The study converted a full-face snorkel mask into a PAPR with the help of cost-effective equipment and 3D-printed adaptors. The result was an 800 g PAPR that was tested according to the NIOSH guidelines. No protection factor was reported for this respirator, and, even though it does not reach the minimum required airflow, it could be proposed as an option to alleviate supply shortages occasionally [116].

## Summary and Recommendations

This paper summarizes different aspects of a Powered Air Purifying Respirator (PAPR), which is a personal dust scrubber. It efficiently captures respirable particulates having sizes exceeding 0.3 $\mu$m. The level of protection depends on the type of mask, the particle’s characteristics, and wearing conditions such as fit, duration, and effort. PAPR’s capture efficacy is quantified in terms of protection factors; a high protection factor implies a better reduction in dust concentration inside the user’s breathing zone. This is calculated as the ratio of the particle concentrations inside and outside the mask. Different protection factors can be calculated depending on the test conditions. Depending on the type of mask, the protection factor can range between 1 (which means no protection) and > 1000. In the case of PAPRs, APFs of 25 for loose-fitting masks and 1000 for tight-fitting masks are considered the minimum. When compared with other types of masks such as the N95, PAPRs can achieve higher levels of protection. PAPRs also offer better comfort in ease of breathing, temperature, and CO_2_ concentration than regular negative-pressure masks. However, other issues have been identified, such as difficulty in communication, the inability to wear other types of equipment along with the mask, and the blower noise. Despite these factors, PAPRs tend to get higher scores in comfort studies compared to other types of masks.

PAPRs are used in several industries where people could be exposed to particulates. The healthcare industry is where the use of these respirators is more common. PAPR usage was prevalent, especially during the SARS-COV-2 pandemic. The mining industry’s inclination towards using PAPRs is attributed to their higher levels of protection against dust particles that can cause serious health problems, including pneumoconiosis. Many mining companies have already petitioned to implement PAPRs in coal mines that meet safety requirements. Other fields where PAPRs are used include the automobile industry, manufacturing operations, lead, and silica bagging. As the use of PAPRs increases, many countries are developing standards and guides to ensure that a minimum level of protection is achieved and that the use of the equipment does not represent a hazard for the workers.

Although CFD simulation is a very useful way of studying the performance of PAPRs, very few such studies exist. However, there are several studies on the evaluation of filters, the main dust-capturing element, using CFD simulations. As PAPRs use a HEPA filter to purify the air, these studies will help understand how dust accumulation, airflow, and other factors alter the efficiency of the filters. These results, then, can be extrapolated to the efficiency of a PAPR. The few studies that use CFD simulation with PAPRs are focused on particle behavior inside of the mask and protection factor prediction. Current research focuses on further development opportunities such as using fans to reduce the CO_2_ concentration inside of the mask. Further research is required to improve these models and to develop test setups. Another study during the SARS-COV-2 pandemic proposed a low-cost model that could be used during high-demand periods to avoid shortage and, therefore, lack of worker protection. This study could be an initial point in the development of new PAPR models that are cheaper than the current ones.

This extensive literature review conclusively establishes PAPR’s ability to supply clean air to the user. Several studies present the efficiency (protection factors) and their comfort while donning the PAPR. Although not required by law, the industry should encourage mine operators to adopt the PAPRs as an additional layer of protection for miners. Although there are very few studies, simulations of the performance of the pleated filters suggest that the HEPA filters used by PAPRs may be able to provide high protection in underground mining environments. The authors recommend more studies on workplace protection factors so that there is industry-specific evidence of the protection provided by the respirators. It is also recommended to develop more CFD simulations for the specific HEPA filters used by PAPRs and under the expected working conditions (e.g., underground mine conditions). The effect of a PAPR on the surroundings of the wearer is also a field that needs further study. Since the blower pulls air from the ambient environment, the behavior of the particles’ transportation around the breathing zone should be studied.

## Data Availability

This review paper used information from publically available sources. They are already cited throughout the manuscript.

## References

[1] Yu H, Zahidi I (2023) Environmental hazards posed by mine dust, and monitoring method of mine dust pollution using remote sensing technologies: an overview. Sci Total Environ 864:161135 36566867 10.1016/j.scitotenv.2022.161135

[2] Bhandari S (2013) Fines and dust generation and control in rock fragmentation by blasting. In: Rock fragmentation by blasting: the 10th international symposium on rock fragmentation by blasting, 2012 (Fragblast 10). Taylor & Francis Books Ltd, pp 511–520

[3] Csavina J, Field J, Taylor MP, Gao S, Landázuri A, Betterton EA, Sáez AE (2012) A review on the importance of metals and metalloids in atmospheric dust and aerosol from mining operations. Sci Total Environ 433:58–73 22766428 10.1016/j.scitotenv.2012.06.013 PMC3418464

[4] Noble TL, Parbhakar-Fox A, Berry RF, Lottermoser B (2017) Mineral dust emissions at metalliferous mine sites. Environ Indicators Metal Mining, 281–306

[5] Timofeeva S, Murzin M (2020) Assessing the environmental risk of mining enterprises by the integral indicator of dust emission. In: IOP conference series: earth and environmental science, vol 408. IOP Publishing, p 012067

[6] Kissell FN (2003) Dust control methods in tunnels and underground mines. Handbook for dust control in mining. US Department of Health and Human Services, Centers for Disease Control and Prevention, National Institute for Occupational Safety and Health, DHHS (NIOSH) Publication, Pittsburgh, PA. (2003-147), 3–21

[7] Sarver E, Keles C, Rezaee M (2019) Beyond conventional metrics: comprehensive characterization of respirable coal mine dust. Int J Coal Geol 207:84–95

[8] Safety O, Administration H et al (2010) Occupational exposure to respirable crystalline silica—review of health effects literature and preliminary quantitative risk assessment. Washington, DC, USA, OSHA

[9] Shekarian Y, Rahimi E, Rezaee M, Su W-C, Roghanchi P (2021) Respirable coal mine dust: a review of respiratory deposition, regulations, and characterization. Minerals 11(7):696

[10] Blackley DJ, Reynolds LE, Short C, Carson R, Storey E, Halldin CN, Laney AS (2018) Progressive massive fibrosis in coal miners from 3 clinics in Virginia. JAMA 319(5):500–501 29411024 10.1001/jama.2017.18444 PMC5839295

[11] Blackley DJ, Halldin CN, Laney AS (2018) Continued increase in prevalence of coal workers’ pneumoconiosis in the United States, 1970–2017. Am J Public Health 108(9):1220–1222 30024799 10.2105/AJPH.2018.304517 PMC6085042

[12] Health MD (2022) Powered Air Purifying Respirator (PAPR)

[13] NIH (2015) The use and effectiveness of powered air purifying respirators in health care: workshop summary. U.S. National Library of Medicine 25996018

[14] Safety O, Administration H et al (2015) Assigned protection factors for the revised respiratory protection standard. Maroon Ebooks

[15] Nelson TJ, Wilmes DP, daRoza RA (1994) Ansi z88. 2 (1992): practices for respiratory protection. Am Ind Hygiene Assoc J 55(7):660–662

[16] Chen Y, Hung S, Kave B, Kluger M, Krieser RB, Lee K, Mezzavia PM, Ng I, Paynter C, Segal R, Sindoni T, Williams DL (2023) HALO CleanSpace PAPR evaluation: communication, respiratory protection, and usability. Infect Control Hosp Epidemiol 44:295–301. 10.1017/ice.2022.71 35361300 10.1017/ice.2022.71 PMC9929704

[17] Cohen HJ, Hecker LH, Mattheis DK, Johnson JS, Biermann AH, Foote KL (2001) Simulated workplace protection factor study of powered air-purifying and supplied air respirators. AIHAJ-Am Ind Hygiene Assoc 62:595–604 10.1080/15298660108984658 11669385

[18] Sekoguchi S, Ando H, Ikegami K, Yoshitake H, Baba H, Ogami A (2022) Measurement of the workplace protection factor of replaceable particulate and powered air-purifying respirators in japanese dust-generating occupations. J UOEH 44:15–24 35249937 10.7888/juoeh.44.15

[19] Nelson TJ, Wheeler TH, Mustard TS (2001) Workplace protection factors—supplied air hood. AIHAJ-Am Ind Hygiene Assoc 62:96–99 10.1080/15298660108984615 11258874

[20] Hyatt EC (1975) Respirator protection factors. Los Alamos National Lab.(LANL), Los Alamos, NM (United States)

[21] Nicas M, Neuhaus J (2004) Variability in respiratory protection and the assigned protection factor. J Occup Environ Hyg 1:99–109 15204884 10.1080/15459620490275821

[22] Bollinger NJ (2004) NIOSH respirator selection logic

[23] NIOSH (2018) A guide to air-purifying respirators. 10.26616/NIOSHPUB2018176

[24] Strickland KT, Bergman MS, Xu S, Zhuang Z (2023) A manikin-based assessment of loose-fitting powered air-purifying respirator performance at variable flow rates and work rates. J Occup Environ Hyg, 1–14 10.1080/15459624.2023.2205481 PMC10527853 37084405

[25] Gao S, McKay RT, Yermakov M, Kim J, Reponen T, He X, Kimura K, Grinshpun SA (2016) Performance of an improperly sized and stretched-out loose-fitting powered air-purifying respirator: manikin-based study. J Occup Environ Hyg 13:169–176 26554716 10.1080/15459624.2015.1098780

[26] Churg A, Wiggs B (1986) Fiber size and number in workers exposed to processed chrysotile asbestos, chrysotile miners, and the general population. Am J Ind Med 9:143–152 3008552 10.1002/ajim.4700090205

[27] Keles C, Sarver E (2022) A study of respirable silica in underground coal mines: particle characteristics. Minerals 12:1555

[28] Colinet J, Halldin CN, Schall J (2021) Best practices for dust control in coal mining

[29] MSHA (2024) MSHA: silica final rule. https://www.msha.gov/sites/default/files/Regulations/Silica-Stakeholder-Meeting-Slides-2024-09-06.pdf

[30] Burgess WA, Shapiro J (1968) Protection from the daughter products of radon through the use of a powered air-purifying respirator. Health Phys 15:115–121 5242156 10.1097/00004032-196808000-00002

[31] Cheberiachko S, Ka OY, Radchuk D, Yavorskyi A (2018) Respiratory protection provided by negative pressure half mask filtering respirators in coal mines. Solid State Phenom 277:232–240

[32] Whitelaw JL, Burton K, Davies B, Jones AL (2019) Respiratory protection: do PAPRs adequately protect workers against DPM?

[33] MSHA (2000) Verification of underground coal mine operators’ dust control plans and compliance sampling for respirable dust. https://www.federalregister.gov/documents/2000/07/07/00-16149/verification-of-underground-coal-mine-operators-dust-control-plans-and-compliance-sampling-for#print

[34] Sapko MJ, Cashdollar KL, Green GM (2007) Coal dust particle size survey of US mines. J Loss Prev Process Ind 20:616–620

[35] Gigliotti S (2021) Petition - Docket No. M-2020-015-C

[36] Gigliotti S (2021) Petition - Docket No. M-2020-016-C

[37] Gigliotti S (2021) Petition - Docket No. M-2019-067-C

[38] Watkins T (2021) Petition - Docket No. M-2019-068-C

[39] Watkins T (2021) Petition - Docket No. M-2019-069-C

[40] Weaver D (2021) Petition - Docket No. M-2021-007-C

[41] Gigliotti S (2021) Petition - Docket No. M-2021-008-C

[42] Watkins T (2022) Petition - Docket No. M-2021-010-C

[43] Watkins T (2022) Petition - Docket No. M-2021-011-C

[44] Watkins T (2022) Petition - Docket No. M-2021-012-C

[45] Watkins T (2022) Petition - Docket No. M-2021-013-C

[46] Watkins T (2022) Petition - Docket No. M-2021-014-C

[47] Watkins T (2022) Petition - Docket No. M-2021-015-C

[48] Watkins T (2022) Petition - Docket No. M-2021-016-C

[49] Watkins T (2022) Petition - Docket No. M-2021-017-C

[50] Watkins T (2022) Petition - Docket No. M-2021-045-C

[51] Carias C, Rainisch G, Shankar M, Adhikari BB, Swerdlow DL, Bower WA, Pillai SK, Meltzer MI, Koonin LM (2015) Potential demand for respirators and surgical masks during a hypothetical influenza pandemic in the United States. Clin Infect Dis 60:42–51 10.1093/cid/civ141 PMC7314226 25878300

[52] Khoo K, Leng P, Ibrahim IB, Lim TK (2005) The changing face of healthcare worker perceptions on powered air-purifying respirators during the SARS outbreak. Respirology 10:107–110 15691247 10.1111/j.1440-1843.2005.00634.x PMC7169158

[53] Licina A, Silvers A, Stuart RL (2020) Use of powered air-purifying respirator (PAPR) by healthcare workers for preventing highly infectious viral diseases—a systematic review of evidence. Syst Rev 9:1–13 32771035 10.1186/s13643-020-01431-5 PMC7414632

[54] Licina A, Silvers A (2021) Use of powered air-purifying respirator (PAPR) as part of protective equipment against sars-cov-2-a narrative review and critical appraisal of evidence. Am J Infect Control 49:492–499 33186678 10.1016/j.ajic.2020.11.009 PMC7654369

[55] Rengasamy S, Eimer BC, Shaffer RE (2009) Comparison of nanoparticle filtration performance of NIOSH-approved and CE-marked particulate filtering facepiece respirators. Ann Occup Hyg 53:117–128 19261695 10.1093/annhyg/men086

[56] Safety U (2025) The meaning of FFP protection classes. https://www.uvex-safety.co.uk/en/meaning-of-ffp-protection-classes/

[57] CDC (2025) NIOSH-approved P100 particulate filtering facepiece respirators. https://shorturl.at/Gh9b6

[58] Penconek A, Drazyk P, Moskal A (2013) Penetration of diesel exhaust particles through commercially available dust half masks. Ann Occup Hyg 57:360–373 23104683 10.1093/annhyg/mes074

[59] Crawford JO, Dixon K, Miller BG, Cherrie JW (2014) A review of the effectiveness of respirators in reducing exposure to polycyclic aromatic hydrocarbons for coke oven workers. Ann Occup Hyg 58:943–954 25053701 10.1093/annhyg/meu048

[60] Spear TM, DuMond J, Lloyd C, Vincent JH (2000) An effective protection factor study of respirators used by primary lead smelter workers. Appl Occup Environ Hyg 15:235–244 10675982 10.1080/104732200301746

[61] Myers WR, Peach III MJ (1983) Performance measurements on a powered air-purifying respirator made during actual field use in a silica bagging operation. Ann Occup Hyg 27:251–259 6314865 10.1093/annhyg/27.3.251

[62] NIOSH (2024) About national institute for occupational safety and health. https://www.cdc.gov/niosh/about/index.html

[63] Service PH, Health D, Services H (2023) 42 CFR Part 84

[64] D’Alessandro M, Schwartz S, Levinson A, Cichowicz JK (2021) Who does what? The Roles of NIOSH, OSHA, and the FDA in respiratory protection in the workplace

[65] Zhao X, Li X, Chai Z, Song H, Kang J (2021) Overview of the application of powered intelligent air-purifying respirator in the health field. IEEE

[66] MSHA (2014) Lowering miners’ exposure to respirable coal mine dust, including continuous personal dust monitors

[67] OSHA (2019) Permissible exposure limits – annotated tables

[68] CDC-NIOSH (2018) Appendix C

[69] OSHA (2019) Permissible exposure limits – annotated tables

[70] Health, Executive S (2002) Wood dust. https://shorturl.at/nbnyy

[71] Heidari MR (2014). N-Methylpyrrolidone. Academic Press. 10.1016/B978-0-12-386454-3.00637-0

[72] EPA US (2024) NAAQS Table. https://www.epa.gov/criteria-air-pollutants/naaqs-table

[73] Roberts V (2014) To PAPR or not to PAPR? Canadian J Resp Therapy: CJRT= Revue canadienne de la therapie respiratoire: RCTR 50:87 PMC4456839 26078617

[74] Schumacher J, Gray SA, Weidelt L, Brinker A, Prior K, Stratling WM (2009) Comparison of powered and conventional air-purifying respirators during simulated resuscitation of casualties contaminated with hazardous substances. Emerg Med J 26:501–505 19546271 10.1136/emj.2008.061531

[75] Sekoguchi S, Shirasaka T, Ando H, Ikegami K, Ogami A (2020) Evaluation of the performance of replaceable particulate and powered air-purifying respirators considering non-recommended wearing methods. Ind Health 58:573–580 32863380 10.2486/indhealth.2020-0056 PMC7708738

[76] Baba H, Ando H, Ikegami K, Sekoguchi S, Shirasaka T, Ogami A (2023) Comparison of respiratory protection during exercise tasks between different methods of wearing replaceable particulate respirators and powered air-purifying respirators. Ind Health 61:275–282 35569997 10.2486/indhealth.2021-0268 PMC10398166

[77] Roberge MR, Vojtko MR, Roberge RJ, Vojtko RJ, Landsittel DP (2008) Wearing an N95 respirator concurrently with a powered air-purifying respirator: effect on protection factor. Respir Care 53:1685–1690 19025703

[78] Nicas M (2024) A critique of occupational safety and health administration’s halfmask respirator assigned protection factor. Annals of the New York Academy of Sciences 10.1111/nyas.15136 38642070

[79] Quinn TD, Sinkule EJ, Goss F (2015) Breathing pressures and inhaled gases of tight- and loose-fitting powered air-purifying respirators: 2963 board #278 may 29, 2: 00 pm - 3: 30 pm. Med Sci Sports Exercise 47

[80] Mackey KRM, Johnson AT, Scott WH, Koh FC (2005) Over breathing a loose-fitting PAPR. Int Soc Resp Protect 22

[81] Berry AR (2015) NIOSH certification standards. https://www.ncbi.nlm.nih.gov/books/NBK294223/

[82] Bostock GJ (1985) An investigation into the performance of positive pressure powered dust hoods and blouses at low flow rates. Ann Occup Hyg 29:415–420 3935031 10.1093/annhyg/29.3.415

[83] Kazakov D, Powe G, Zhu J (2022) An innovative approach to evaluating protection level of tight-fitting PAPRs when face seal is compromised. J Int Soc Resp Protect 39

[84] Alderman TS, Stiegel MA, Estes RA, Thomann WR, Sempowski GD (2016) Field-testing method for loose-fitting powered air-purifying respirators equipped with HEPA filters. Appl Biosafety 21:71–78

[85] Janssen L, Bidwell J, Cuta K, Nelson T (2008) Workplace performance of a hood-style supplied-air respirator. J Occup Environ Hyg 5:438–443 18464097 10.1080/15459620802115930

[86] Rhee MSM, Lindquist CD, Silvestrini MT, Chan AC, Ong JJY, Sharma VK (2021) Carbon dioxide increases with face masks but remains below short-term NIOSH limits. BMC Infect Dis 21:1–7 33858372 10.1186/s12879-021-06056-0 PMC8049746

[87] Weiss R, Guchlerner L, Weissgerber T, Filmann N, Haake B, Zacharowski K, Wolf T, Wicker S, Kempf VAJ, Ciesek S (2021) Powered air-purifying respirators used during the SARS-CoV-2 pandemic significantly reduce speech perception. J Occupation Med Toxicology 16:1–11 10.1186/s12995-021-00334-y PMC8481762 34592994

[88] Kempfle JS, Panda A, Hottin M, Vinik K, Kozin ED, Ito CJ, Remenschneider AK (2021) Effect of powered air-purifying respirators on speech recognition among health care workers. Otolaryngol Head Neck Surg 164:87–90 32689877 10.1177/0194599820945685

[89] Heating G (2025) What does natural gas smell like? https://getzschman.com/blog/what-does-natural-gas-smell-like/

[90] Xia X, Liu S, Xia K, Liu Y, Zhang J, Liu X, Yao Y, Li G (2022) The effect of wearing a powered air purifying respirator versus an N95 mask on the olfactory function of healthcare workers 10.1097/MD.0000000000032669 PMC9857538 36701701

[91] Sinkule EJ, Powell JB, Rubinstein EN, McWilliams L, Quinn T, Pugliese M (2016) Physiologic effects from using tight-and loose-fitting powered air-purifying respirators on inhaled gases, peak pressures, and inhalation temperatures during rest and exercise. J Int Soc Resp Protect 33:36 PMC7183739 32336877

[92] Meh D, Denišlič M (1994) Quantitative assessment of thermal and pain sensitivity. J Neurol Sci 127:164–169 7707075 10.1016/0022-510x(94)90069-8

[93] Wang Z, Li S, Ren T, Wu J, Lin H, Shuang H (2019) Respirable dust pollution characteristics within an underground heading face driven with continuous miner – a CFD modeling approach. J Clean Prod 217:267–283. 10.1016/j.jclepro.2019.01.273

[94] Biswal PK, Parida D, Mishra G, Sahoo AK (2019) Study of air flow pattern in mine model gallery and its validation using CFD modelling. World Sci News 130:1–24

[95] Grabowski J, Jurtz N, Brandt V, Kruggel-Emden H, Kraume M (2023) Comparison of sub-grid drag laws for modeling fluidized beds with the coarse grain DEM-CFD approach. Computational Particle Mech, 1–20

[96] Dedering M, Stausberg W, Iliev O, Lakdawala Z, Ciegis R, Starikovicius V (2008) On new challenges for CFD simulation in filtration

[97] Boris JP (2005) On large eddy simulation using subgrid turbulence models comment 1. Springer

[98] Wang W, Li J (2007) Simulation of gas-solid two-phase flow by a multi-scale CFD approach—of the emms model to the sub-grid level. Chem Eng Sci 62:208–231

[99] Myers SH, Walters DK (2005) A one-dimensional subgrid near wall treatment forturbulent flow CFD simulation. 42193:577–585

[100] Elrahmani A, Al-Raoush RI, Abugazia H, Seers T (2022) Pore-scale simulation of fine particles migration in porous media using coupled CFD-DEM. Powder Technol 398:117130

[101] Čiegis R, Iliev O, Lakdawala Z (2007) On parallel numerical algorithms for simulating industrial filtration problems. Comput Methods Appl Math 7:118–134

[102] Cheberiachko SI, Slavinskyi DV, Cheberiachko YI, Deryugin OV (2023) Mathematical model of air flow movement in a motorized filter respirator. Natsional’nyi Hirnychyi Universytet. Naukovyi Visnyk, 97–103. 10.33271/nvngu/2023-3/097

[103] Cagna M, Boehle M (2002) Application of CFD methods for the simulation of the flow through a filter in dependency of the operating time. 36150:1013–1017

[104] Fotovati S, Hosseini SA, Tafreshi HV, Pourdeyhimi B (2011) Modeling instantaneous pressure drop of pleated thin filter media during dust loading. Chem Eng Sci 66:4036–4046

[105] Li A, Ahmadi G (1992) Dispersion and deposition of spherical particles from point sources in a turbulent channel flow. Aerosol Sci Technol 16:209–226

[106] Feng Z, Long Z, Chen Q (2014) Assessment of various CFD models for predicting airflow and pressure drop through pleated filter system. Build Environ 75:132–141

[107] Tronville P, Sala R (2003) Minimization of resistance in pleated-media air filter designs: empirical and CFD approaches. HVAC&R Res 9:95–106

[108] Yue C, Zhang Q, Zhai Z (2016) Numerical simulation of the filtration process in fibrous filters using CFD-DEM method. J Aerosol Sci 101:174–187. 10.1016/j.jaerosci.2016.08.004

[109] Xu SS, Lei Z, Zhuang Z, Bergman M (2019) Computational fluid dynamics simulation of flow of exhaled particles from powered-air purifying respirators. Am Soc Mech Eng 10.1115/detc2019-97826 PMC10193452 37216194

[110] Koh FC, Johnson AT, Rehak TE (2011) Inward leakage in tight-fitting PAPRs. J Environ Public Health 2011 10.1155/2011/473143 PMC3103902 21647352

[111] Bergman M, Lei Z, Xu S, Strickland K, Zhuang Z (2019) Validation of computational fluid dynamics models for evaluating loose-fitting powered air-purifying respirators. Springer 10.1007/978-3-319-96089-0_20 PMC10658266 37987021

[112] Yuasa H, Kumita M, Honda T, Kimura K, Nozaki K, Emi H, Otani Y (2015) Breathing simulator of workers for respirator performance test. Ind Health 53:124–131 25382381 10.2486/indhealth.2014-0079 PMC4380599

[113] Zhu J, He X, Bergman MS, Guffey S, Nimbarte AD, Zhuang Z (2019) A pilot study of minimum operational flow for loose-fitting powered air-purifying respirators used in healthcare cleaning services. J Occupation Environ Hygiene 10.1080/15459624.2019.1605241 PMC6720108 31081727

[114] Zhang X, Li H, Shen S, Rao Y, Chen F (2016) An improved FFR design with a ventilation fan: CFD simulation and validation. PLoS ONE 11:0159848 10.1371/journal.pone.0159848 PMC4959710 27454123

[115] Birgersson E, Tang EH, Lee WLJ, Sak KJ (2015) Reduction of carbon dioxide in filtering facepiece respirators with an active-venting system: a computational study. PLoS ONE 10:0130306 10.1371/journal.pone.0130306 PMC4482734 26115090

[116] Khoo D, Yen C-C, Chow WT, Jain P, Loh N-HW, Teo WW, Koh C (2020) Ultra-portable low-cost improvised powered air-purifying respirator: feasibility study. Br J Anaesth 125:264–266 10.1016/j.bja.2020.04.082 PMC7203042 32446500

